# Paraneoplastic Diarrhea From Medullary Thyroid Carcinoma Resolved With Yttrium-90 Radioembolization of Liver Metastases

**DOI:** 10.1210/jcemcr/luae103

**Published:** 2024-07-29

**Authors:** Sarah Hamidi, Armeen Mahvash, Mimi I Hu

**Affiliations:** Department of Endocrine Neoplasia and Hormonal Disorders, The University of Texas MD Anderson Cancer, Houston, TX 77030, USA; Department of Interventional Radiology, The University of Texas MD Anderson Cancer, Houston, TX 77030, USA; Department of Endocrine Neoplasia and Hormonal Disorders, The University of Texas MD Anderson Cancer, Houston, TX 77030, USA

**Keywords:** medullary thyroid carcinoma, liver metastases, radioembolization, Yttrium-90, paraneoplastic diarrhea

## Abstract

Medullary thyroid carcinoma (MTC) can often have an indolent course despite distant metastatic disease. Additionally, given that metastatic MTC is incurable and systemic therapies have non-negligeable toxicities, localized treatments are often favored in presence of oligo-progressive disease. Transarterial radioembolization (TARE) with yttrium-90 (Y90) has emerged as a safe and efficacious treatment for nonresectable primary and metastatic liver tumors, yet data supporting its use in metastatic MTC are limited. We present the case of a patient with hereditary MTC and large bilobar liver metastases who demonstrated tumor response and resolution of their paraneoplastic diarrhea following TARE with Y90 microspheres.

## Introduction

Medullary thyroid carcinoma (MTC) is a rare neuroendocrine tumor originating from the parafollicular C cells of the thyroid and represents only 1% to 2% of all thyroid cancers [1]. Up to 20% of patients present with distant metastases, most commonly in the lungs, liver, and bones [2, 3]. Although many patients can have an indolent course despite distant disease, the 10-year survival from time of first metastasis is 10% to 40% [1]. High-burden disease may also lead to paraneoplastic diarrhea, which can be debilitating and is often refractory to antimotility agents. Although incompletely understood, MTC-related diarrhea is thought to result from tumoral secretion of various substances, including calcitonin, prostaglandins, and/or serotonin [4]. Somatostatin analogs achieve only modest symptom improvement, while tumor debulking, by reducing the humoral secretion of pro-diarrheal substances, is often effective in alleviating the diarrheal syndrome [2, 5, 6].

Liver metastases (LM) are present in up to 45% of patients with advanced MTC. Treatment is indicated when they are large, progressive, and/or symptomatic. In some instances, LM can be isolated and treated with localized therapies such as surgical resection or radiofrequency ablation. However, in most cases they are multiple and disseminated throughout the parenchyma. In this situation, chemoembolization or systemic therapy are recommended [2]. Because metastatic MTC is incurable, loco-regional therapies are often favored over systemics in presence of oligo-metastatic disease. Transarterial chemoembolization (TACE) of LM from MTC has been shown to provide durable radiological and symptomatic control, although reported response rates are variable [7-9]. While TACE with drug-eluting beads allows less systemic toxicity compared to conventional TACE, it may come with an increased risk for liver necrosis [10, 11].

Over the past decade, transarterial radioembolization (TARE) with yttrium-90 (Y90) microspheres has emerged as a novel therapy for primary and secondary liver malignancies. However, limited data are available regarding its safety and efficacy for the treatment of LM from MTC. Here we report the case of a patient with hereditary MTC and multifocal LM who experienced significant radiological and symptomatic improvement following TARE with Y90.

## Case Presentation

A 31-year-old female patient was diagnosed with stage IVC MTC and found to carry a germline *RET* C620R mutation. At initial presentation, she had innumerable lung and bilobar liver metastases, in addition to extensive cervical lymphadenopathy in the central and lateral compartments. The largest liver lesion measured 2.9 cm in segment VIII. Her baseline calcitonin level was 41 968 pg/mL (41 968 ng/L) (reference: ≤ 7.6 pg/mL; ≤7.6 ng/L), and carcinoembryonic antigen (CEA) level was 502.9 ng/mL (502.9 µg/L) (reference: 0.0–3.8 ng/mL; 0.0–3.8 µg/L). She underwent a total thyroidectomy with bilateral central and lateral neck dissections in November 2019.

## Diagnostic Assessment

**Figure 1.**
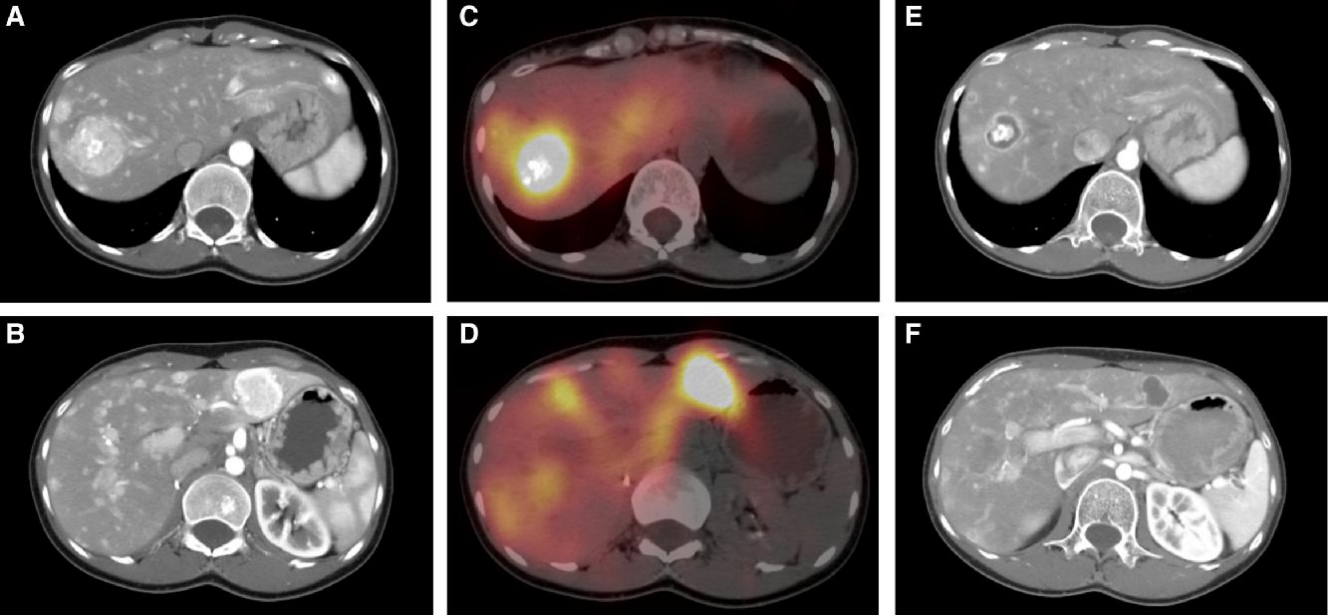
Liver metastases before (A and B), immediately after (C and D) and 5 months after (E and F) transarterial radioembolization with Y90-SIR-spheres. SPECT/CT images (C and D) show significant radiation emission from dominant liver metastases immediately following therapy.

The patient's distant disease was then watched for several years without any additional treatment, with very slow progression. In August 2023, the largest liver lesion measured 3.8 × 4.1 cm (Fig. 1A and 1B). She also had oligo-progressive disease in a dominant 1.9 cm right lower lobe lung metastasis, while the rest of the pulmonary and osseous lesions remained stable. Doubling times for calcitonin and CEA were relatively slow, at 2.7 and 3.7 years respectively. Nonetheless, the patient had significant diarrhea with associated weight loss, despite antimotility drugs, which warranted intervention.

## Treatment

Although initiating a selective RET inhibitor could have been reasonable given the uncontrolled diarrhea and burdensome metastatic disease, the patient wanted to avoid long-term systemic therapy for personal reasons. It was thus decided to favor localized therapies with external beam radiation therapy (EBRT) to the oligo-progressive lung nodule and Y90-TARE of the LM. Pretreatment calcitonin level was 97 081 pg/mL (97 081 ng/L), CEA was 575 ng/mL (575 µg/L), and liver function tests were normal.

Prior to TARE, the patient underwent a planning hepatic angiography with in-room computed tomography (CT) angiography, and a technetium 99m-labeled macroaggregated albumin (^99m^Tc-MAA) injection followed by MAA single-photon emission computed tomography (SPECT)/CT which showed multifocal tumor MAA localization and a lung shunt percentage of 3.2%. Treatment planning was performed using the partition dosimetry model. Bilobar LM were present, and the total liver volume was 1485 cc^3^ with a tumor burden of 220 cc^3^ (15%). The treatment plan for administration of 31 mCi of resin microspheres (Sirtex Medical) divided between the liver lobes was performed and delivered successfully. Post-Y90 SPECT/CT imaging (Fig. 1C and 1D) confirmed mean tumor dose of 150 Gy and mean normal liver dose of 18 Gy. The patient was discharged home on the same day as treatment and no short-term complications occurred.

## Outcome and Follow-Up

**Figure 2.**
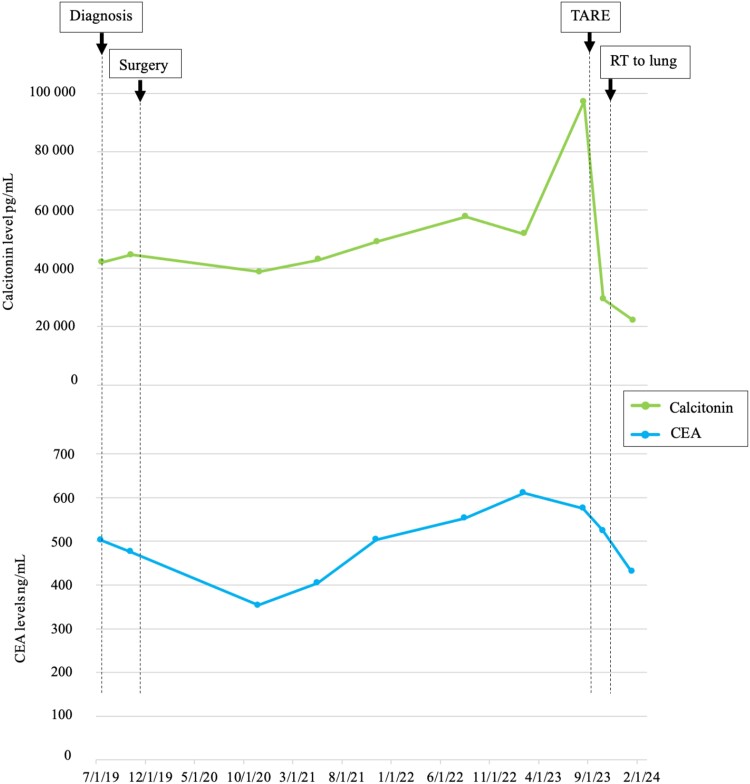
Evolution of patient's tumor markers. Abbreviations: TARE, transarterial radioembolization; RT, radiation therapy. Calcitonin and CEA levels are equivalent in conventional units (pg/mL, ng/mL) and SI units (ng/L, µg/L).

At subsequent restaging 5 months later, the patient had significant tumor shrinkage in the treated LM: for example, the dominant segment VII/VIII mass was reduced from 3.8 cm to 2.4 cm, while another left lesion was reduced from 3.4 cm to 2.2 cm (Fig. 1E and 1F). There was also significant decrease in calcitonin of more than 50%, and in CEA by 25% (Fig. 2). Most importantly, about a month after undergoing radioembolization, the patient experienced complete resolution of her diarrhea and no longer required antimotility agents for control. Of note, she underwent EBRT to the lung 2 months after TARE, at which time the diarrhea had already resolved, suggesting that resolution of the diarrhea was achieved exclusively by the treatment of LM with Y90. Following these localized therapies, the patient's other sites of disease remained stable, so that we were able to continue with active surveillance and defer initiating systemic therapy.

## Discussion

Many patients with metastatic MTC will have an indolent clinical course, with slowly progressive disease. Additionally, none of the currently available systemic therapies provide cure, and these drugs have non-negligeable potential adverse effects. Even the better tolerated RET inhibitor, selpercatinib, has a risk for hypertension and elevation of liver transaminases, among other adverse effects [12]. In patients with progressive disease that could be contributing to difficult-to-control symptoms, systemic therapy may be considered; however, patients should also be offered other focal therapeutic options, especially when there is oligo-progression [2, 13-15]. Multifocal LM are frequent in advanced MTC and are more often associated with paraneoplastic diarrhea compared with other sites of distant disease [2]. Although TACE of LM has led to durable clinical responses in some patients with MTC, it has variable efficacy. Grozinsky-Glasber et al reported a 100% partial response rate in 7 patients with LM from MTC treated with TACE, with a median time to progression of 38 months [8]. On the other hand, Fromingé et al treated 12 patients with TACE and reported a partial radiological tumor response in only 5/12 (42%), and improvement of diarrhea in 2/5 (40%) [7]. Beyond the variable response rates, chemoembolization has several other limitations. While there is a rationale for combining the embolic particle with a chemotherapeutic agent to which the tumor is sensitive, no cytotoxic chemotherapy has been shown to provide significant benefits in metastatic MTC. Moreover, there is a risk for systemic toxicity if the chemotherapeutic agent penetrates the systemic circulation, although this is minimized with drug-eluting bead (DEB)-TACE [10, 16].

Over the past 20 years, Y90-TARE has emerged as a novel therapy for primary liver carcinoma and LM from various solid tumors, with significant radiological and clinical benefit, and relatively low toxicity [17-19]. Y90 is a pure beta emitter with limited tissue penetration and a short half-life, making it an ideal transarterial liver-targeting agent. Its mean tissue penetration is less than 2.5 mm, allowing selective high-dose irradiation of the tumor tissue while sparing the adjacent normal liver parenchyma. The Y90-labeled microspheres have a diameter of 25 to 35 micrometers, allowing them to be delivered to the tumor via the hepatic artery, without passing into the venous circulation. This is crucial, as hepatic tumors derive most of their blood supply from the arterial hepatic circulation, whereas the normal liver is predominantly fed by the portal veinous circulation. Because of their size, the microspheres will be trapped within the arteriolar vasculature surrounding the tumor, leading to selective radiation-induced tumor necrosis [20]. Two Y90 microsphere products are commercially available and FDA-approved: the glass-based TheraSphere™ and the resin-based SIR-Spheres®. In hepatocellular carcinoma, some data suggest longer time to tumor progression with TARE compared to TACE [21, 22].

Y90-TARE allows to effectively treat patients with significant tumor burden, often in a single outpatient treatment session, as opposed to classic TACE, which requires multiple sessions. Compared with chemoembolization, TARE also tends to cause less arterial ischemia because of the smaller particle size and microembolic technique. Thus, Y90-TARE is generally well tolerated and causes limited liver injury, especially when candidates are carefully selected and advanced dosimetry is utilized [23]. Optimal candidates for planned whole liver treatment should have normal baseline total bilirubin and albumin levels. Several additional measures mitigate the risk of complications: the use of cone beam CT and SPECT/CT for treatment planning, which is mandatory by international guidelines, reduces the risk of extrahepatic complications [23], while limiting the normal liver dose to less than 40 Gy and using a treatment planning software reduce the risk of hepatic decompensation. Inadequate tumor dose based on the planning phase examination, and extrahepatic microsphere deposition that cannot be mitigated with current techniques, are exclusion criteria to Y90-TARE. Potential side effects include: a mild postembolization syndrome with constitutional symptoms and abdominal pain; hepatic dysfunction, more frequent in patients with cirrhosis or those exposed to cytotoxic chemotherapy within the previous 2 months; hepatic fibrosis and/or portal hypertension; and lymphopenia [20, 24, 25]. Rarely, penetration of the Y90 microspheres into the gastrointestinal vascular bed can lead to gastric or duodenal ulcers, while significant hepatopulmonary shunting can cause radiation-induced pneumonitis [24]. Thus, an absolute contraindication to TARE is significant shunting to the lungs or gastrointestinal tract, as assessed by a pretreatment ^99m^Tc-MAA scan. Other relative contraindications include prior radiation therapy to the liver, and Child-Pugh class C cirrhosis.

Our case illustrates the potential of Y90 microspheres for the treatment of LM from MTC, allowing marked improvement in paraneoplastic diarrhea through tumor debulking and decrease in calcitonin levels, and delay in systemic therapy initiation. After a short-term follow-up of 5 months, no immediate adverse events had occurred in our otherwise young healthy patient with a normal baseline liver function. To the best of our knowledge, this is the first report of a case in which TARE with Y90 led to resolution of paraneoplastic diarrhea in a patient with advanced MTC.

Only one study has been published demonstrating the feasibility and efficacy of TARE with Y90-labeled microspheres for the treatment of LM from MTC [26]. In this study, 8 patients with liver-dominant metastatic sporadic MTC, who had progressive and/or symptomatic disease and < 50% liver involvement, were treated with Y90-resin microspheres. Among the 6 patients who completed the 18 months of follow-up, 1 achieved a complete response, 4 had a partial response, and 1 patient had stable disease. All patients had decreases in calcitonin and CEA levels, and none of them needed to start systemic therapy after 18 months. No information was provided about paraneoplastic diarrhea improvement post-TARE. The 6 patients had only mild to moderate elevations in their liver enzymes, which were transient in most cases. However, 1 patient who was excluded from the analysis had a severe liver injury, although it had resolved 3 months later. This was the only patient in whom both hepatic lobes were treated at the same time.

In conclusion, Y90 radioembolization for LM from MTC seems to result in a favorable radiologic and biochemical response, potentially allowing to delay the initiation of systemic therapy in patients with oligo-progressive disease in the liver. Additionally, TARE seems to achieve sufficient tumor debulking to significantly reduce the secretion of pro-diarrheal humoral substances and relieve paraneoplastic diarrhea. Nevertheless, although generally well tolerated, this procedure can lead to significant hepatic and extrahepatic toxicity if patients are not carefully selected and if treatment is not planned appropriately. Additional data are needed to determine the long-term efficacy and potential complications of radioembolization of LM from MTC with Y90-labeled microspheres.

## Learning Points

- Transarterial radioembolization (TARE) with yttrium-90 (Y90) microspheres is to be considered among localized therapy options for oligo-progressive and/or symptomatic liver metastases from medullary thyroid carcinoma (MTC).
- Our case illustrates the efficacy of Y90 microspheres in achieving significant tumor shrinkage and reduction in calcitonin levels in a patient with large bilobar liver metastases from MTC, leading to resolution of paraneoplastic diarrhea and allowing for delay in systemic therapy initiation.
- Careful patient selection and treatment planning with advanced dosimetry are crucial to avoid hepatic and extrahepatic toxicity from Y90-TARE.

## Contributors

All authors made individual contributions to authorship. S.H.: conceptualization; literature review; data curation; writing—original draft. A.M.: supervision; validation; writing—review and editing; preparation of radiology images; patient care. M.I.H.: supervision; validation; writing—review and editing; patient care. All authors reviewed and approved the final draft.

## Data Availability

Data sharing is not applicable to this article as no datasets were generated or analyzed during the current study.

## Abbreviations

CEA: carcinoembryonic antigen
CT: computed tomography
EBRT: external beam radiation
LM: liver metastases
MAA: macroaggregated albumin
MTC: medullary thyroid carcinoma
SPECT/CT: single-photon emission computed tomography/CT
TACE: transarterial chemoembolization
TARE: transarterial radioembolization; Y90, yttrium-90

## References

[1] Angelousi A, Hayes AR, Chatzellis E, Kaltsas GA, Grossman AB. Metastatic medullary thyroid carcinoma: a new way forward. Endocr Relat Cancer. 2022;29(7):R85‐R103. 35521769 10.1530/ERC-21-0368 PMC9175549

[2] Wells SA, Jr., Asa SL, Dralle H, et al Revised American thyroid association guidelines for the management of medullary thyroid carcinoma. Thyroid. 2015;25(6):567‐610. 25810047 10.1089/thy.2014.0335 PMC4490627

[3] Matrone A, Gambale C, Prete A, Elisei R. Sporadic medullary thyroid carcinoma: towards a precision medicine. Front Endocrinol (Lausanne). 2022;13:864253. 35422765 10.3389/fendo.2022.864253 PMC9004483

[4] Shakir MKM, Spiro AJ, Mai VQ, Hoang TD. Diarrhea as an initial presentation in patients with medullary thyroid cancer: delaying the diagnosis. Case Rep Gastroenterol. 2020;14(2):391‐401. 32884516 10.1159/000508850 PMC7443648

[5] Gild ML, Clifton-Bligh RJ, Wirth LJ, Robinson BG. Medullary thyroid cancer: updates and challenges. Endocr Rev. 2023;44(5):934‐946. 37204852 10.1210/endrev/bnad013 PMC10656709

[6] Vainas I, Koussis C, Pazaitou-Panayiotou K, et al Somatostatin receptor expression in vivo and response to somatostatin analog therapy with or without other antineoplastic treatments in advanced medullary thyroid carcinoma. J Exp Clin Cancer Res. 2004;23(4):549‐559. 15743023

[7] Fromigué J, De Baere T, Baudin E, Dromain C, Leboulleux S, Schlumberger M. Chemoembolization for liver metastases from medullary thyroid carcinoma. J Clin Endocrinol Metab. 2006;91(7):2496‐2499. 16608897 10.1210/jc.2005-2401

[8] Grozinsky-Glasberg S, Bloom AI, Lev-Cohain N, Klimov A, Besiso H, Gross DJ. The role of hepatic trans-arterial chemoembolization in metastatic medullary thyroid carcinoma: a specialist center experience and review of the literature. Eur J Endocrinol. 2017;176(4):463‐470. 28100632 10.1530/EJE-16-0960

[9] Hughes P, Healy NA, Grant C, Ryan JM. Treatment of hepatic metastases from medullary thyroid cancer with transarterial embolisation. Eur Radiol Exp. 2017;1(1):9. 29708149 10.1186/s41747-017-0013-6 PMC5909341

[10] Song JE, Kim DY. Conventional vs drug-eluting beads transarterial chemoembolization for hepatocellular carcinoma. World J Hepatol. 2017;9(18):808‐814. 28706579 10.4254/wjh.v9.i18.808 PMC5491403

[11] Guiu B, Deschamps F, Aho S, et al Liver/biliary injuries following chemoembolisation of endocrine tumours and hepatocellular carcinoma: lipiodol vs. drug-eluting beads. J Hepatol. 2012;56(3):609‐617. 22027582 10.1016/j.jhep.2011.09.012

[12] Hadoux J, Elisei R, Brose MS, et al Phase 3 trial of selpercatinib in advanced RET-mutant medullary thyroid cancer. N Engl J Med. 2023;389(20):1851‐1861. 37870969 10.1056/NEJMoa2309719

[13] Cabanillas ME, Ryder M, Jimenez C. Targeted therapy for advanced thyroid cancer: kinase inhibitors and beyond. Endocr Rev. 2019;40(6):1573‐1604. 31322645 10.1210/er.2019-00007 PMC7341904

[14] Laurie SA, Banerji S, Blais N, et al Canadian consensus: oligoprogressive, pseudoprogressive, and oligometastatic non-small-cell lung cancer. Curr Oncol. 2019;26(1):e81‐e93. 30853813 10.3747/co.26.4116 PMC6380642

[15] Nguyen KT, Sakthivel G, Milano MT, Qiu H, Singh DP. Oligoprogression in non-small cell lung cancer: a narrative review. J Thorac Dis. 2022;14(12):4998‐5011. 36647502 10.21037/jtd-22-536 PMC9840049

[16] Razi M, Jianping G, Xu H, Ahmed MJ. Conventional versus drug-eluting bead transarterial chemoembolization: a better option for treatment of unresectable hepatocellular carcinoma. J Interv Med. 2021;4(1):11‐14. 34805941 10.1016/j.jimed.2020.10.006 PMC8562211

[17] Raval M, Bande D, Pillai AK, et al Yttrium-90 radioembolization of hepatic metastases from colorectal cancer. Front Oncol. 2014;4:120. 25120951 10.3389/fonc.2014.00120 PMC4110696

[18] Salem R, Johnson GE, Kim E, et al Yttrium-90 radioembolization for the treatment of solitary, unresectable HCC: the LEGACY study. Hepatology. 2021;74(5):2342‐2352. 33739462 10.1002/hep.31819 PMC8596669

[19] Tsang ES, Loree JM, Davies JM, et al Efficacy and prognostic factors for Y-90 radioembolization (Y-90) in metastatic neuroendocrine tumors with liver metastases. Can J Gastroenterol Hepatol. 2020;2020:5104082. 33299824 10.1155/2020/5104082 PMC7704205

[20] Ahmadzadehfar H, Biersack HJ, Ezziddin S. Radioembolization of liver tumors with yttrium-90 microspheres. Semin Nucl Med. Mar. 2010;40(2):105‐121. 10.1053/j.semnuclmed.2009.11.001 20113679

[21] Salem R, Gordon AC, Mouli S, et al Y90 radioembolization significantly prolongs time to progression compared with chemoembolization in patients with hepatocellular carcinoma. Gastroenterology. 2016;151(6):1155‐1163.e2. 27575820 10.1053/j.gastro.2016.08.029 PMC5124387

[22] Dhondt E, Lambert B, Hermie L, et al (90)Y radioembolization versus drug-eluting bead chemoembolization for unresectable hepatocellular carcinoma: results from the TRACE phase II randomized controlled trial. Radiology. 2022;303(3):699‐710. 35258371 10.1148/radiol.211806

[23] Levillain H, Bagni O, Deroose CM, et al International recommendations for personalised selective internal radiation therapy of primary and metastatic liver diseases with yttrium-90 resin microspheres. Eur J Nucl Med Mol Imaging. 2021;48(5):1570‐1584. 33433699 10.1007/s00259-020-05163-5 PMC8113219

[24] Tong AK, Kao YH, Too CW, Chin KF, Ng DC, Chow PK. Yttrium-90 hepatic radioembolization: clinical review and current techniques in interventional radiology and personalized dosimetry. Br J Radiol. 2016;89(1062):20150943. 26943239 10.1259/bjr.20150943 PMC5258157

[25] Carr BI, Metes DM. Peripheral blood lymphocyte depletion after hepatic arterial 90Yttrium microsphere therapy for hepatocellular carcinoma. Int J Radiat Oncol Biol Phys. 2012;82(3):1179‐1184. 21601995 10.1016/j.ijrobp.2010.10.042

[26] Puleo L, Agate L, Bargellini I, et al Yttrium-90 transarterial radioembolization for liver metastases from medullary thyroid cancer. Eur Thyroid J. 2022;11(6):e220130. 36126186 10.1530/ETJ-22-0130 PMC9641787

